# Modelling Lyssavirus Infections in Human Stem Cell-Derived Neural Cultures

**DOI:** 10.3390/v12040359

**Published:** 2020-03-25

**Authors:** Vinod Sundaramoorthy, Nathan Godde, Ryan J. Farr, Diane Green, John M. Haynes, John Bingham, Carmel M. O’Brien, Megan Dearnley

**Affiliations:** 1 Commonwealth Scientific and Industrial Research Organisation (CSIRO), Australian Animal Health Laboratory (AAHL), East Geelong, VIC 3219, Australia; vinod.sundaramoorthy@csiro.au (V.S.); nathan.godde@csiro.au (N.G.); ryan.farr@csiro.au (R.J.F.); diane.green@csiro.au (D.G.); john.bingham@csiro.au (J.B.); 2 Monash Institute of Pharmaceutical Sciences, 399 Royal Parade, Parkville, VIC 3052, Australia; john.haynes@monash.edu; 3 CSIRO Manufacturing, Research Way, Clayton, VIC 3168, Australia; 4 Australian Regenerative Medicine Institute, Monash University, Clayton, VIC 3168, Australia

**Keywords:** rabies, lyssavirus, stem cell-derived neurons, ex-vivo models, viral pathogenesis, trans-synaptic axonal trafficking, chemokine and cytokine response, neuronal apoptosis

## Abstract

Rabies is a zoonotic neurological infection caused by lyssavirus that continues to result in devastating loss of human life. Many aspects of rabies pathogenesis in human neurons are not well understood. Lack of appropriate ex-vivo models for studying rabies infection in human neurons has contributed to this knowledge gap. In this study, we utilize advances in stem cell technology to characterize rabies infection in human stem cell-derived neurons. We show key cellular features of rabies infection in our human neural cultures, including upregulation of inflammatory chemokines, lack of neuronal apoptosis, and axonal transmission of viruses in neuronal networks. In addition, we highlight specific differences in cellular pathogenesis between laboratory-adapted and field strain lyssavirus. This study therefore defines the first stem cell-derived ex-vivo model system to study rabies pathogenesis in human neurons. This new model system demonstrates the potential for enabling an increased understanding of molecular mechanisms in human rabies, which could lead to improved control methods.

## 1. Introduction

The genus *Lyssavirus,* belonging to family *Rhabdoviridae*, consists of a group of negative strand RNA viruses capable of causing rabies-like disease in humans and other mammals [1]. Rabies virus belongs to the prototype *Rabies lyssavirus* species and usually transmitted between terrestrial carnivore species, to a lesser extent bat species and humans [2]. In addition, there are 15 other officially recognized species within the *Lyssavirus* genus, most of which have been isolated from bats [1,3,4]. Although effective vaccination is available for post-exposure prophylaxis (PEP), rabies still persists as a global health issue, accounting for a significant number of preventable deaths [5,6,7]. The death rate from rabies is particularly high in developing countries due to poor access to PEP and many of the victims are children under the age of 15 years [6,7,8].

Victims are usually infected through the bite of infected animals. The virus then invades the neuromuscular junctions at the site of bite entering the host nervous system [5]. Once inside the neuronal axons, the virus uses anterograde trafficking to spread through the nervous system until it reaches the brain [9,10], resulting in encephalitis. Viral infections in the central nervous system (CNS) present significant challenges due to its separation from the peripheral immune system by the blood brain barrier (BBB). CNS resident cells, including neurons, secrete a variety of chemokines to stimulate infiltration of immune cells into the CNS for effective viral clearance [11,12,13,14,15], which is aided by immune-modulatory cells such as microglia and astrocytes [16,17]. However, such immune response is highly ineffective in clearing rabies virus [18]. This is due to the ability of rabies virus to inhibit innate and adaptive immune responses using diverse strategies, including inhibition of interferon response by phosphoprotein [19,20,21] and virus-induced apoptosis of immune T cells [22,23,24]. While immune-mediated viral clearance is impeded in rabies infection, there is upregulation of specific chemokines and cytokines, linked with inflammation and enhancement of blood-brain barrier permeability, which could be detrimental to the nervous system [25,26,27,28]. In addition, rabies infection does not typically result in the loss of neurons or neuronal apoptosis in the nervous system [29,30]. In fact, efficient rabies infection restricts neuronal apoptosis to enhance viral replication in neurons [18,31]. Despite the lack of neuronal loss, rabies infection causes severe neurological dysfunction in the host, resulting in clinical features such as paralysis, behavioral, and cognitive deficits [32,33,34]. This indicates that rabies infection could induce neuronal dysfunction rather than killing the neurons. However, these key pathogenic mechanisms in rabies are yet to be examined in ex-vivo models of human neurons.

Studies on rabies pathogenesis to date have predominantly relied on animal models. Mouse models and primary mouse neuronal cultures have been the preferred source to investigate rabies pathogenesis in-vivo and in-vitro, respectively. These model systems have identified several key pathogenic mechanisms, which have greatly improved our understanding of rabies [5,10,35,36,37]. However, there are ethical and practical constraints which can limit the investigation of upstream neuronal pathogenic mechanisms in living animals and in primary neurons. Ideally, development of a human neuronal model system, which could be used in concert with current animal models, would greatly assist the investigation of viral pathogenesis in humans. In addition, a species-specific model system would significantly contribute towards better understanding features of viral pathogenesis in human infection, such as viral adaptations at a molecular level that enable host-switching for efficient infection in the human nervous system. While there have been previous attempts to utilize such human neuronal model systems [38,39], stem cell technologies have not yet been explored to model rabies pathogenesis. Such technologies could provide an ethically renewable, high throughput platform for screening potential therapeutics against human rabies.

The advancement of stem cell technology has greatly enabled the generation of ex-vivo models of human CNS for studying neurodegenerative diseases and infections. This includes successful modelling of neurotropic viral infections such as herpes simplex virus (HSV) [40], varicella zoster virus (VSV) [41], La Crosse virus [42] and flaviviruses such as Zika virus [43,44], West Nile virus [45], dengue virus [44,46], and Japanese encephalitis virus (JEV) [47] in stem cell-derived human neural cultures. Such advances are enabled by efficient protocols for specific differentiation of embryonic stem cells (ESC) and induced pluripotent stem cells (iPSC) into functional neural lineage cultures representative of CNS resident cells [48]. Although ESC and iPSC have similar differentiation capacity [49,50], differences in origin may influence neuronal modelling [51,52,53,54,55,56] and susceptibility to viral infections [57]. Hence, investigation into disease modelling using stem cell-derived neural cultures should consider the differences in ESC or iPSC origin of these model systems.

In this study, we generated and characterized human stem cell-derived neural model systems for the study of rabies pathogenesis. We differentiated human ESC- and iPSC-derived neuronal precursor cells (hNPCs) into forebrain-type neural cultures consisting of mature neurons and astrocytes. Using these neural cultures, we studied the pathogenesis of different lyssavirus strains, including laboratory-adapted CVS-11 rabies virus and several field strains of lyssavirus isolated from infected dogs or bats. In these studies, we demonstrate efficient infection of both ESC- and iPSC-derived human neurons with all virus strains. We next examined whether these ex-vivo models consisting of stem cell-derived human neurons would reproduce key pathogenic mechanisms associated with rabies. We show that, consistent with the existing knowledge of rabies pathogenesis, lyssavirus infection in human neural cell cultures did not result in the activation of neuronal apoptosis. However, infection with CVS-11 and a field rabies virus strain resulted in the upregulation of a specific subset of cytokines and chemokines involved in enhancing BBB permeability and neuroinflammation. Additionally, we generated an ex-vivo model using microfluidics to study the trans-synaptic trafficking of rabies virus along the human neuronal network. This novel ex-vivo model system demonstrated axonal trafficking of rabies virus in human neuronal network and highlighted differences in axonal transmission dynamics between laboratory-adapted CVS-11 and a field rabies virus strain. This describes a novel ex-vivo model system in which to undertake a controlled study of axonal trafficking of rabies virus between interconnected human neurons. In summary, this study reports the first detailed characterization of lyssaviruses infection of in-vitro differentiated human neural cultures. This could serve as a new model system for future studies of the biological and immunological responses of human neurons to lyssaviruses, complimenting the existing in-vivo and in-vitro animal models of rabies.

## 2. Materials and Methods

### 2.1. Ethics Statement

Human ethics: All work using human iPSC and ESC pluripotent cell (hPSC) lines and their derivative neural lineage cell types, was carried out in accordance with Australia’s National Health and Medical Research Council (NHMRC) ‘National Statement on Ethical Conduct in Human Research’ (2007, updated 2018), the ‘Australian Code for the Responsible Conduct of Research’ (2007, updated 2018), and with approvals from the Monash University Human Research Ethics Committee for parental pluripotent cell lines (20060009880 04/11/2006, 2015000129 04/02/2015), and from the Commonwealth Scientific and Industrial Research Organization (CSIRO) Health and Medical Research Ethics Committee for human neural precursor cell lines (LR 16/2017 29/11/2017).

Animal ethics: All experiments involving suckling mouse for rabies virus amplification in this study were reviewed and approved by the Animal Ethics Committee (AEC) of the Australian Animal Health Laboratory (AAHL) (approval #1605, 05/04/2013), following the Australian National Health and Medical Research Council Code of Practice for the Care and Use of Animals for Scientific Purposes.

### 2.2. Cell Culture

#### 2.2.1. Generation of Human Neural Precursor Cells (hNPCs)

The iPSC line HDF51i-509, previously derived by Sendai reprogramming of human dermal fibroblast (HDF) cells [58], was kindly provided under materials transfer agreement by Prof. Jeanne Loring (The Scripps Research Institute, La Jolla, CA, USA). The hESC line WA09 [59] (H9), was provided under materials transfer agreement with WiCell Research Institute, Madison, WI, USA, http://www.wicell.org/. Differentiation of parental HDF51i-509 and H9 pluripotent cell (hPSC) lines to neural stem and progenitor precursors (hNPCs) was performed, as described previously [60]. Briefly, hPSCs maintained in Essential 8^TM^ (E8) medium on Geltrex^TM^-coated tissue cultureware [61] were harvested with Accutase (Thermofisher Scientific, Waltham, MA, USA) and transferred to AggreWell^TM^ 800 culture plates (STEMCELL Technologies, Vancouver, BC, Canada) for the formation of uniform aggregate embryoid bodies (EBs) [62], in serum-free NeuroCult^TM^ NS-A human Basal Medium supplemented with, 20 ng/mL human recombinant bFGF, 20 ng/mL human recombinant EGF (Peprotech, Rocky Hill, NJ, USA), and 500 ng/mL human noggin (R&D systems, Minneapolis, MN) [63,64,65,66]. EBs were transferred following 5 days of the above neural lineage induction culture to ultra-low attachment culture plates (Corning, NY, USA) for neurosphere induction in the same medium conditions. Neurosphere suspension cultures were expanded for a further 9 days with medium replenished every 2 days, then dissociated and seeded to tissue cultureware coated at 1 µg/cm^2^ with Englebreth-Holm-Swarm murine sarcoma basement membrane laminin (Sigma-Aldrich, St. Louis, MO, USA, 10µg/mL in 1:1 DMEM/Hams F12 (DMEM/F12), Thermofisher Scientific, Waltham, MA, USA), expanding the hNPCs as monolayer cultures in the above mitogenic media without noggin supplement [67,68]. Renewing neural stem cell cultures were transferred after three passages to STEMdiff^TM^ Neural Progenitor Medium (NPM), (STEMCELL Technologies, Vancouver, BC, Canada) for adherent maintenance culture. Established hNPC lines were successfully maintained from each parental derivation for up to 15 passages in STEMdiff^TM^ NPM, cryopreserved in STEMdiff^TM^ NPM Freeze Medium, and thawed as an expanding population of hNPCs.

#### 2.2.2. Differentiation of hNPCs

HDF51i-509-NPCs and H9-NPCs were differentiated in-vitro according to an adapted protocol (STEMCELL Technologies, Vancouver, BC, Canada) for neural lineage induction by mitogen withdrawal [69] and maturation in a physiological neuronal basal medium [70]. Briefly, hNPCs were seeded at 15,000–25,000 cells/cm^2^ in 24 well tissue culture plates coated with 15 µg/mL poly-L-ornithine (Sigma-Aldrich, St. Louis, MO, USA) in phosphate buffered saline (PBS) and 10 µg/mL laminin (Sigma-Aldrich, St. Louis, MO, USA) in DMEM/F12 (1µg/cm^2^, Thermofisher Scientific, Waltham, MA, USA), then expanded for ~2 days in STEMDiff^TM^ NPM at 37 °C in an atmosphere of 5% CO_2_ in air until at 40–50% confluency. Neural differentiation was initiated by replacing 50% *v*/*v* of the culture medium with BrainPhys^TM^ neural medium supplemented with 2% *v*/*v* Neurocult^TM^ SM1 Neuronal Supplement, 1% *v*/*v* N2 Supplement-A (STEMCELL Technologies, Vancouver, BC, Canada), 20 ng/mL human recombinant brain-derived neurotrophic factor (BDNF), 20 ng/mL human recombinant glial cell line-derived neurotrophic factor (GDNF) (Peprotech, Rocky Hill, NJ, USA), 1 mM dibutyrl cyclic-AMP sodium salt (Sigma-Aldrich, St. Louis, MO, USA), 200 nM ascorbic acid (STEMCELL Technologies, Vancouver, BC, Canada), making complete BrainPhys^TM^ neural medium. HNPC differentiation and neural maturation was continued for up to 35 days with 50% *v*/*v* complete BrainPhys^TM^ media changes each 2–3 days.

### 2.3. Calcium Imaging

Calcium imaging was performed on both ESC- and iPSC-derived neural cultures as described previously [71], after at least 21 days of hNPC differentiation. Briefly, neural cultures were loaded, in media, with 5 μM Fura-2 AM (ABCAM, Cambridge, UK) for 30–40 min at 37 °C under 5% CO_2_ in air. For imaging, cultures were incubated with HEPES buffered salt solution (consisting of NaCl, 145 mM; KCl, 5 mM; MgSO_4_, 1 mM; HEPES, 10 mM; CaCl_2_, 2 mM; glucose, 10 mM, containing 0.1% bovine serum albumin, at pH 7.4) and imaged using Nikon TE2000U microscope (Nikon, Minato City, Tokyo, Japan) with 20× objective and a glass stage heated to 37 °C. A DG4 (Sutter, Novato, CA, USA) was used to illuminate cells with light at 340 and 380 nm. Fluorescence emission at 510 nm was recorded every 1–2 s. Cells morphologically characteristic of neurons, with fine axonal projections, were selected as regions of interest (ROI) for intensity measurement and analysis. Background emission was subtracted from each image and 340/380 ratios of the resultant intensity emission values at each time point were obtained. Drugs such as ATP, glutamate, or dopamine (Sigma-Aldrich, St. Louis, MO, USA) were added to the cell cultures at 100 µm during imaging to stimulate calcium influx. The cultures were washed with HEPES buffer between each drug treatment and allowed enough time for cells to reach basal excitation state before subsequent stimulation.

### 2.4. Virus Stock Amplification and Quantification

Laboratory-adapted CVS-11 strain was passaged and amplified in Neuro-2a cells (ATCC: CCL131, Manassas, VA, USA). Original lyssavirus field strains such as an Australian bat lyssavirus strain isolated from an infected horse (H.ABLV), a Canadian silver-head bat rabies virus (SHBRV), and a rabies virus strain isolated from an infected dog in Zimbabwe (Z.Dog) were amplified by intracerebral inoculation in suckling mouse brains (Swiss mice). After this, 10% mouse brain homogenates in 0.05 M phosphate buffered saline (PBSA) containing the virus were used to infect Neuro-2a cells for further amplification. The viral strains were subjected to fewer than 3 passages in Neuro-2a cells during amplification. The viral titer of cell culture supernatants were determined by titration assays on BHK-21 cells (CSL, Parkville, Vic, Australia) by direct fluorescent antibody test. Briefly, serial 10-fold dilutions of viral suspensions were prepared in cell culture media and were added to 96 well plates (4 replicates each), followed by BHK cell suspensions. The cells were then incubated at 37 °C with 5% CO_2_ for 5–6 days. The plates were fixed with 10% formalin for 30 min at room temperature and then stained with FITC conjugated anti-rabies monoclonal antibody (Fujirebio, Tokyo, Japan) at 1:10 dilution in 0.5% *w*/*v* bovine serum albumin (BSA)/phosphate buffered saline (PBSA) with 0.005% Evans blue. Plates were read with an Olympus BX51 inverted microscope (Olympus, Shinjuku City, Tokyo, Japan) and the median tissue culture infectious dose (TCID_50_) determined [72]. The titer values of viral stocks used in this study were in the range of 10^5–6^ TCID_50_/mL. All experiments involving viruses were performed in the biosafety level 3 (BSL3) laboratories at Australian Animal Health Laboratory (AAHL), following protocols approved by AAHL’s institutional biosafety committee.

### 2.5. Viral Infection of Human ESC- and iPSC-Derived Neural Cultures

For infection, hPSC-derived neural cultures in 24 well plates were treated with appropriate volumes of viral inoculum required to infect at virus: cell ratio of 1 (based on the viral titre determined in BHK-21 cells and the number of hNPCs seeded in each well prior to differentiation, i.e., ~40,000 cells per well). For infecting hPSC-derived neural cultures in microfluidic chambers, media from the panel to be inoculated was removed and the appropriate volume of viral inoculum required to infect at a virus:cell ratio of 1 was added (based on the number of hNPCs seeded in the panel, i.e., 80,000 cells). A unidirectional flow of media was always maintained with a higher volume of media in the non-inoculated panel (200 µL) and a lower volume in the inoculated panel (100 µL).

### 2.6. Generation of Trans-Synaptic Human Neural Cultures Using Microfluidics

To generate an ex-vivo model of interconnected human neurons, xona microfluidic devices (XONA Microfluidics, Cat#SND150, NC, USA) mounted on glass coverslips (24 × 40 mm; Menzel Glaser, Braunschweig, Germany) coated with poly-L-ornithine (Sigma-Aldrich, St. Louis, MO, USA) were used. HDF51i-509-NPCs were seeded in both panels of a microfluidic device at a density of 80,000 cells per panel. After 2–3 days of further expansion in StemDiff NPM medium, differentiation was initiated by changing the media in the wells with BrainPhys^TM^ complete neural differentiation media as described earlier. The hNPCs were then allowed to differentiate for 3–4 weeks with media change in the wells every 2 days. After differentiation and infection experiments, neural cultures were fixed in the chamber with 4% *w*/*v* paraformaldehyde (PFA, Sigma-Aldrich, St. Louis, MO, USA) in PBSA for 1 h, followed by immunostaining and confocal imaging in the chamber.

### 2.7. Immunocytochemistry

Human stem cell-derived neural cultures cultured on coverslips were fixed with 4% *w*/*v* PFA (Sigma-Aldrich, St. Louis, MO, USA) in PBSA for 1 h at room temperature. The coverslips were washed gently three times with PBSA and the cells were permeabilized with 0.1% *v*/*v* Triton X-100 (Sigma-Aldrich, St. Louis, MO, USA) in PBSA for 5 min, rinsed with PBSA, then blocked with 0.5% *w*/*v* BSA in PBSA for 30 min and incubated overnight at 4 °C with primary antibodies diluted in 0.5% *w*/*v* BSA in PBSA. They were washed three times with PBSA and incubated with species-specific fluorescent secondary antibodies (Alexa Fluor, Thermofisher Scientific, Waltham, MA, USA) diluted at 1:200 in 0.5% *w*/*v* BSA in PBSA for 1 h at room temperature. Coverslips were rinsed twice with PBSA, twice with sterile water, and stained with 4′,6-diamidino-2-phenylindole (DAPI) for 10 min, then were washed twice with sterile water and mounted on glass slides (Thermofisher Scientific Waltham, MA, USA) with Vectashield mounting medium (Vector Laboratories, Burlingame, CA, USA).

The following primary antibodies were used at the indicated dilutions: chicken anti-MAP2 (1:1000, ABCAM, Cambridge, UK, cat#ab4674), rabbit anti-GFAP (1:1000, DAKO, Berlin, Germany cat#Z0334), mouse anti-beta tubulin III/TUJ1 (1:1000, ABCAM, Cambridge, UK, cat#ab78078), rabbit anti-rabies nucleoprotein, 1:3000, in-house [73], mouse pan-axonal neurofilament antibody (1:1000, BioLegend, San Diego, CA, USA, cat#837904), and mouse anti-NeuN (1:100, Merck, Kenilworth, NJ, USA, Cat#MAB377).

### 2.8. Apoptosis (TUNEL) Assay

To quantify apoptosis in human stem cell-derived neural cultures infected with rabies virus, terminal deoxynucleotidyl transferase dUTP nick end labeling (TUNEL) staining was performed according to manufacturer’s protocol using the in-situ cell death detection kit, TMR red (Sigma-Aldrich, St. Louis, MO, USA, cat#12 156 792 910). As a positive control for DNA fragmentation, fixed and permeabilized neuronal cultures on coverslips were treated with 3 units of DNaseI (Thermofisher Scientific, Waltham, MA, USA) in 50 mM Tris-HCL for 15 min to induce DNA strand breaks. The cells were then stained with pan-axonal neurofilament antibody to identify neurons. Confocal images of neurons stained with TUNEL TMR red, neurofilament and DAPI were taken with 20× objective covering at least 100 neurons per image. The percentage of TUNEL-positive apoptotic bodies in these images (threshold normalized to controls), relative to DAPI stained nuclei were counted using imageJ (NIH, LOCI, University of Wisconsin, Madison, WI, USA) particle analyzer plugin and watershed function, with the following parameters: size (inch^2^)—0.003-infinity and circularity—0.00–1.00.

### 2.9. Confocal Imaging

Confocal imaging was performed using a ZEISS LSM 800 (ZEISS, Oberkochen, BW, Germany) inverted confocal microscope. Images were taken as Z-stacks and then maximum intensity projection was generated. For imaging neuronal network in microfluidic devices, a tile imaging was performed covering an area of 2.04 mm × 892.84 µm with Z-stacks (39 slices; 39.9 µm) and then stitched together for maximum intensity projection. All the confocal imaging and processing were performed using ZEN 2.5 Blue software (ZEISS, Oberkochen, BW, Germany).

### 2.10. RNA Extraction and cDNA Generation

Total RNA from human stem cell-derived neural cultures infected with rabies virus strains were extracted using miRCURY RNA Isolation Kit—Cell and Plant (Exiqon, Copenhagen, Denmark) following manufacturer’s protocol. RNA concentration was measured using a Nanodrop ND-1000 spectrophotometer (Thermofisher Scientific, Waltham, MA, USA) and the purity was confirmed by an absorbance ratio A260/A280 of 2.0–2.1. cDNA was synthesized from RNA using SuperScirpt^TM^ III First-Strand synthesis system (Thermofisher Scientific, Waltham, MA, USA) following manufacturer’s protocol. Random hexamers provided with the kit was used for non-specific reverse transcription of all RNA species.

### 2.11. qPCR for Chemokine and Cytokine Gene Expression Analysis

qPCR analysis was performed for chemokine and cytokine genes using a 96-well panel of primers for use with SYBR Green (BIO-RAD, Hercules, CA, USA, cytokines and chemokines, SAB target list, Human). qPCR analysis was performed using this panel of primers for a total of 91 human chemokines and cytokines following manufacturer’s protocol using an ABI QuantStudio^TM^ 6 Flex Real-Time PCR instrument (Applied Biosystems, Foster city, CA, USA), in a 96-well FAST reaction, with the threshold for all reactions set to 0.1. Cycling conditions were as follows: 95 °C for 20 s followed by 40 cycles of 95 °C for 0.01 s and 60 °C for 20 s. A selection of 29 genes was identified to be differentially expressed in the initial screen on ESC-derived neural cultures infected with CVS-11 rabies virus. Then, a customized 96-well panel of primers was designed (BIO-RAD, Hercules, CA, USA) for the 29 genes, which was then used to perform qPCR analysis on both ESC- and iPSC-derived neural cultures infected with two rabies virus strains (CVS-11 and Z.Dog). Each reaction contained 10 ng cDNA. Results were normalized to β-actin expression (included in the same reaction) and then converted to relative abundance via the fold-over-detectable method [74] using a Ct value of 40 as the detectability cut-off. Any undetectable results are reported with Ct of 40.

### 2.12. qPCR for Apoptosis and Necrosis Markers

RNA was isolated and reverse transcribed using TRizol and SuperScirpt^TM^ III reagents (Thermofisher Scientific, Waltham, MA, USA) following manufacturer’s instructions using random hexamers. cDNA amplification was performed using a Quantstudio™ 6 Flex Real-time PCR system (Applied Biosystems, Foster city, CA, USA), in a 384-well standard reaction, with threshold for all reactions was set to 0.1. Cycling conditions were as follows: 50 °C for 2 min, 95 °C for 10 min, followed by 40 cycles of 95 °C for 15 s and 60 °C for 1 min. Each reaction contained 20 ng cDNA. Samples were normalized to GAPDH for samples from H9-NPCs-derived neural cultures, ACTB for samples from HDF51i-509-NPC-derived neural cultures and expression levels calculated using the fold-over-detectable method [74]. Real-time primers included ACTB-F ACCATGGATGATGATATCGC, ACTB-R TCATTGTAGAAGGTGTGGTG, GAPDH-F AATGAAGGGGTCATTGATGG, GAPDH-R AAGGTGAAGGTCGGAGTCAA, MAP2-F CCACCTGAGATTAAGGATCA, MAP2-R GGCTTACTTTGCTTCTCTGA, GFAP-F GTACCAGGACCTGCTCAAT, GFAP-R CAACTATCCTGCTTCTGCTC, RIP1-F CATGGAAAAGGCGTGATACAC, RIP1-R ACTTCCCTCAGCTCATTGTG, caspase9-F TGCTGAGCAGCGAGCTGTT, caspase9-R AGCCTGCCCGCTGGAT.

### 2.13. qPCR Assays for Detection of Lyssavirus

Lyssavirus nucleoprotein RNA and 18S ribosomal (r)RNA were measured using AgPath-ID One-Step RT-PCR kit (Applied Biosystems, Foster city, CA, USA) as per the manufacturer’s instructions, with a total RNA input of 30 ng. RABVD1 primers and FAM probe [75] were used detect CVS-11, SHBRV and Z.Dog strains, while the insectivorous ABLV primers and FAM probe [76] were used to detect H.ABLV. Both RABVD1 and ABLV primers were synthesized by Integrated DNA Technologies. Eukaryotic 18S rRNA Endogenous Control (VIC™ / MGB probe, primer limited) primer/probe mix (Applied Biosystems, Foster city, CA, USA) was used for the detection of 18S rRNA. Quantitative PCR was conducted on the QuantStudio™ 6 Flex Real-Time PCR instrument (Applied Biosystems, Foster city, CA, USA) using the standard reaction. Cycling conditions were as follows: 50 °C for 2 min, 95 °C for 10 min, followed by 40 cycles of 95 °C for 15 s and 60 °C for 1 min. The threshold for all reactions was set to 0.1. Lyssavirus nucleoprotein RNA levels were normalized to 18S rRNA. Any undetectable results are reported with Ct of 40.

### 2.14. Statistical Analysis

Changes in cytokine and chemokine expression were tested using the non-parametric Kruskal–Wallis H test. Any mRNA that rejected the null hypothesis (*p* < 0.05) was subjected to a post-hoc Dunn’s Multiple Comparison Test with Benjamini–Krieger–Yekutieli two-stage false discovery rate correction [77]. These comparisons were completed using the SciPy [78] and scikit-posthocs [79] Python packages. Statistical significance between two values was determined using a two-tailed t test using Prism 7 (GraphPad, San Diego, CA, USA).

Data are presented as mean ± SEM. * *p* < 0.05, ** *p* < 0.01, **** *p* < 0.0001. *n* = 3, indicates three independent experiments.

## 3. Results

### 3.1. Generation and Characterization of Human ESC- and iPSC-Derived Neural Cultures

**Figure 1:**
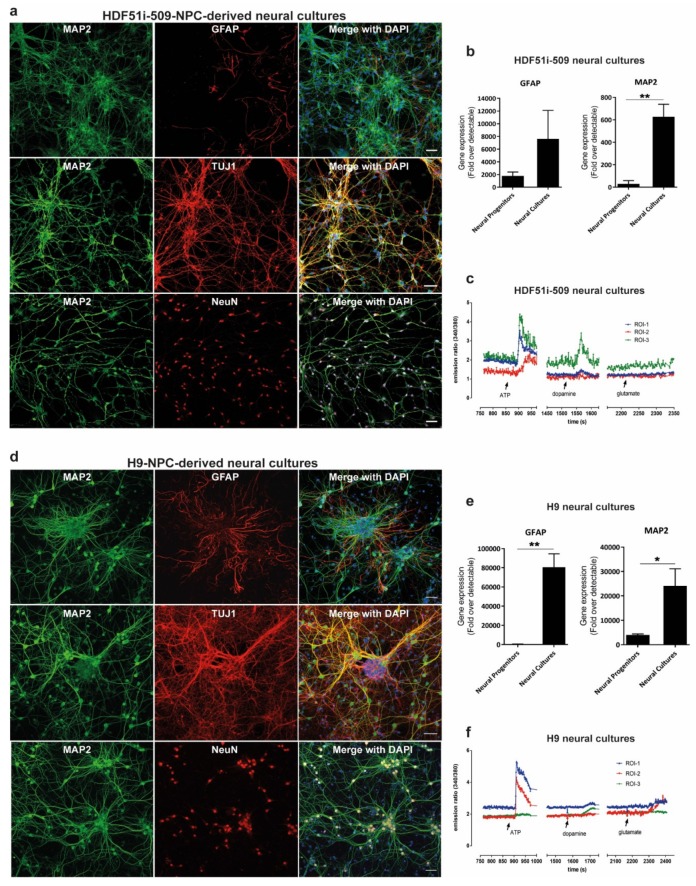
Generation of forebrain type human neural lineage cell cultures from induced pluripotent stem cells (iPSC)- and embryonic stem cells (ESC)-derived neuronal precursor cells (hNPCs). (**a**) HDF51i-509-derived and (**d**) H9-derived hNPCs were differentiated into forebrain type human neural cell cultures for 24 days and 21 days, respectively. The cultures were then immunostained for the detection of mature neuron markers MAP2, beta tubulin III (TUJI), and NeuN, as well as astrocytes using anti-GFAP antibody. Images were taken using 20× objective. Scale bar 50 µm (images are maximum intensity projection of Z-stacks). Gene expression of neuronal (MAP2) and glial (GFAP), markers detected by qPCR in (**b**) HDF51i-509-derived and (**e**) H9-derived cultures following differentiation. ** *p* < 0.01, * *p* < 0.05 neural progenitors vs neural cultures, *n* = 3. (**c**) HDF51i-509-NPC and (**f**) H9-NPC-derived neural cultures were subjected to live calcium imaging following loading with the calcium binding dye, Fura-2 AM. Cells morphologically characteristic of neurons with axonal projections are identified as regions of interest (ROI) for Fura-2 intensity measurements. Graphs (**c**,**f**) show representative calcium influx (measured by ratio of emission intensities at 340 and 380 nm) in three different ROI in response to treatment with 100 µM ATP, 100 µM glutamate and 100 µM dopamine. Arrows indicate the timepoint at which stimulants were added.

In order to study rabies pathogenesis, we generated human neural cultures from the parental HDF51i-509-iPSC [58] and WA09(H9)-ESC [59] lines. Renewing neuronal precursor cell (hNPC) lines [60] were first established from each parental cell line. HNPCs were then further differentiated in-vitro, as described previously [69,70], to establish mature forebrain-type neural cultures. These H9-NPC and HDF51i-509-NPC cultures were fixed following 21 and 24 days of differentiation respectively, for characterization by immunostaining and confocal microscopy. This demonstrated that both ESC- and iPSC-derived neural cultures consisted of mature neurons which positively stained for MAP2, TUJI (beta tubulin III) and NEUN, with GFAP-positive astrocytes (Figure 1a,d. To confirm efficient neural induction, we also performed gene expression analysis for neuronal (MAP2) and glial (GFAP) markers using qPCR (Figure 1b,e). This analysis showed greater expression of MAP2 and GFAP genes in differentiated neural cultures compared to the corresponding hNPC lines, demonstrating differentiation into neurons and astrocytes. To further evaluate the development of neuronal signaling mechanisms in these neural cultures, we performed calcium imaging with Fura-2 AM dye following 21 days and 26 days of differentiation of H9-NPC and HDF51i-509-NPC cultures, respectively. Calcium imaging demonstrated robust calcium influx in both these neural cultures in response to pharmacological stimulation with ATP and lower levels of calcium influx was recorded upon stimulation with dopamine or glutamate in some but not all neuronal cells imaged (Figure 1c,f, Supplementary Video S1). This suggests the presence of a mixed population of CNS resident neurons with varied excitability in response to stimulation with common neurotransmitters. In summary, these results demonstrate that both the H9 and HDF51i-509 derived neural cultures generated in this study consisted of mature and functional neurons with supporting astrocytes, representing a model neural culture system representative of human CNS resident cells.

### 3.2. Lyssavirus Infection of Human Stem Cell-Derived Neural Cultures

**Figure 2:**
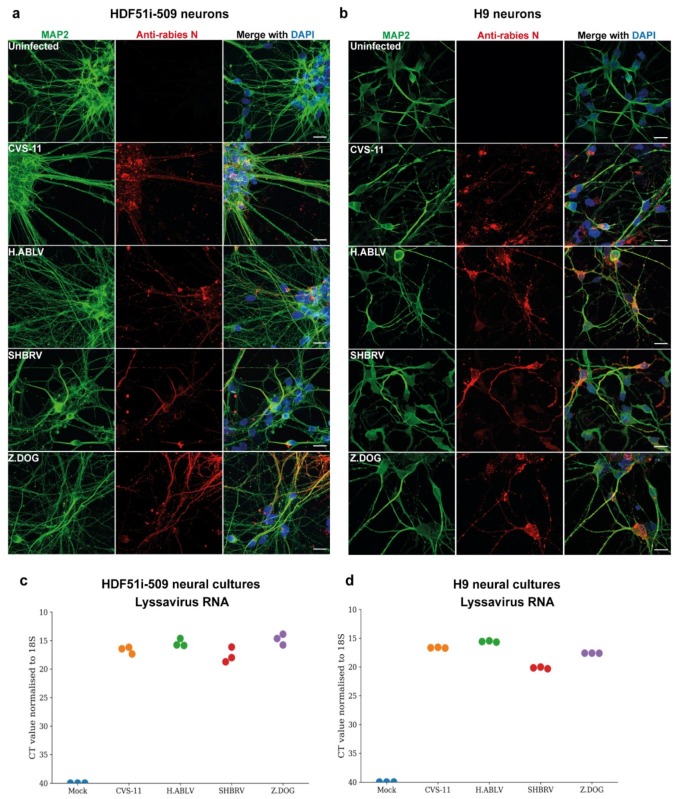
Lyssavirus infection of stem cell-derived human neural cultures. (**a**) HDF51i-509-NPCs and (**b**) H9-NPCs were differentiated for 21 days and 29 days, respectively. Differentiated neural cultures were then infected with different lyssavirus strains (CVS-11, H.ABLV, SHBRV and Z.DOG) at a virus:cell ratio of 1 for 72 h. Representative images show infection of (**a**) HDF51i-509-NPC-derived and (**b**) H9-NPC-derived neural cultures immunostained for detection of MAP2-positive neurons (green) and rabies virus antigen by anti-nucleoprotein staining (red). Nuclei stained with DAPI (blue), shown as a merged image with red and green channels. Scale bar 50 µm (images are maximum intensity projection of Z-stacks). (**c**,**d**) Validation of viral infection shown by qPCR analysis of lyssavirus RNA in (**c**) HDF51i-509-NPC- and (**d**) H9-NPC-derived neural cultures, *n* = 3.

To examine the susceptibility of H9- and HDF51i-509-derived neural cultures to infection with different lyssavirus strains, cells were infected with laboratory-adapted CVS-11 strain, an Australian bat lyssavirus strain isolated from an infected horse (H.ABLV), a Canadian silver-head bat rabies virus (SHBRV), and a rabies virus strain isolated from an infected dog in Zimbabwe (Z.Dog). The neural cultures were infected with viral strains at a virus:cell ratio of 1 and examined for infectivity at 72 h post-infection. Immunostaining analysis demonstrated effective infection of both H9- and HDF51i-509-derived neural cultures with all the lyssavirus strains (Figure 2a,b). This resulted in the detection of rabies nucleoprotein antigen in the cell body as well as neurites of MAP2-positive neurons. However, no cytopathic effect was observed in the neural cultures infected with all the lyssavirus strains. In addition, qPCR analysis was performed using specific primers for the amplification and detection of nucleoprotein RNA from each of the rabies virus strains. Such an analysis detected high level of lyssavirus RNA in these neural cultures, indicating efficient infection (Figure 2c,d).

### 3.3. Lack of Apoptosis in Human Stem Cell-Derived Neural Cultures Infected with Lyssavirus

**Figure 3:**
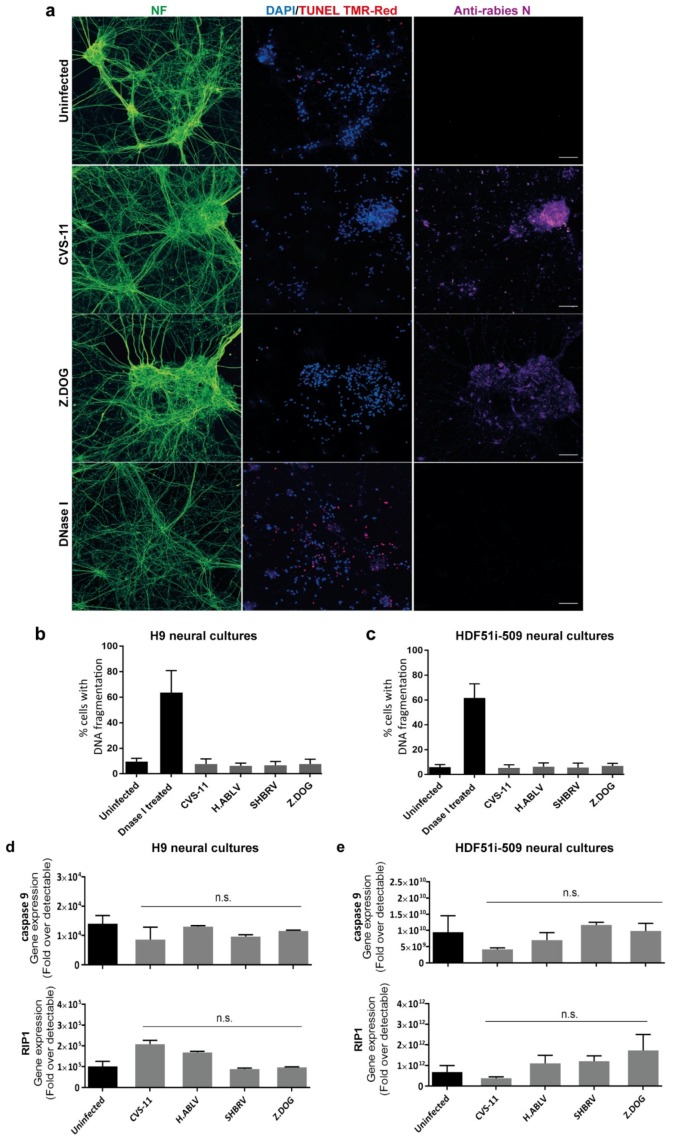
Lack of apoptosis in stem cell-derived human neural cultures infected with lyssavirus strains. HDF51i-509-NPCs and H9-NPCs were differentiated for 24 days and 29 days, respectively. These neural cultures were infected with different lyssavirus strains for 72 h at virus:cell ratio of 1 and examined for apoptosis by TUNEL staining (TMR-red). (**a**) Representatives images of HDF51i-509-NPC-derived neurons identified by anti-neurofilament immunofluorescence (NF, green) stained with TUNEL (red) and DAPI (blue) for nuclei. Infection with each lyssavirus strain was identified by anti-nucleoprotein staining (magenta). Images were taken with 20× objective and are maximum intensity projections of Z-stacks. Scale bar 50 µm. Quantification of apoptotic DNA fragmentation using TUNEL staining in (**b**) H9-NPC-derived neurons and (**c**) HDF51i-509-NPC-derived neurons. At least 500 neurons were quantified per sample, *n* = 3. No statistical significance in apoptotic cell death observed between uninfected and lyssavirus infected neurons. qPCR analysis of apoptotic (caspase9) and necrosis (RIP1) genes in total RNA from (**d**) H9-NPC-derived and (**e**) HDF51i-509-NPC-derived neural cell cultures. No statistical significance difference is observed in the expression of caspase9 and RIP1 genes in uninfected and lyssavirus infected neural cultures.

Since human stem cell-derived neural cultures were highly susceptible to lyssavirus infection, we next examined whether such an infection could result in the induction of DNA fragmentation, indicative of neuronal apoptosis by TUNEL staining. DNA fragmentation was specifically examined in neurons identified by pan-axonal neurofilament antibody (Figure 3a), excluding the analysis of glial cells present in these neural cultures. This analysis revealed that none of the lyssavirus strain infection induced any significant increase in neuronal DNA fragmentation as compared to uninfected neural cultures derived from both H9 (Figure 3b) and HDF51i-509 (Figure 3c). However, a significantly higher percentage of TUNEL positive neurons were observed upon treatment with DNaseI enzyme, which induces DNA fragmentation reminiscent of apoptosis (Figure 3b,c). In addition, qPCR analysis was performed on the total RNA extracted from the lyssavirus infected neural cultures using specific primers for apoptotic and necrosis markers (Figure 3d,e). Such an analysis included RNA from both neuronal and glial cells in these cultures. This revealed negligible transcriptional activation of key genes involved in apoptosis (caspase9) or necrosis (RIP1) [80] in infected compared to non-infected neural cultures. This shows that lyssavirus infection does not induce apoptosis and cell death in human neural cultures containing both neuronal and glial cell types.

### 3.4. Upregulation of Distinct Pro-Inflammatory Chemokine and Cytokines in Human Stem Cell-Derived Neural Cultures during Infection with Rabies Virus

**Figure 4:**
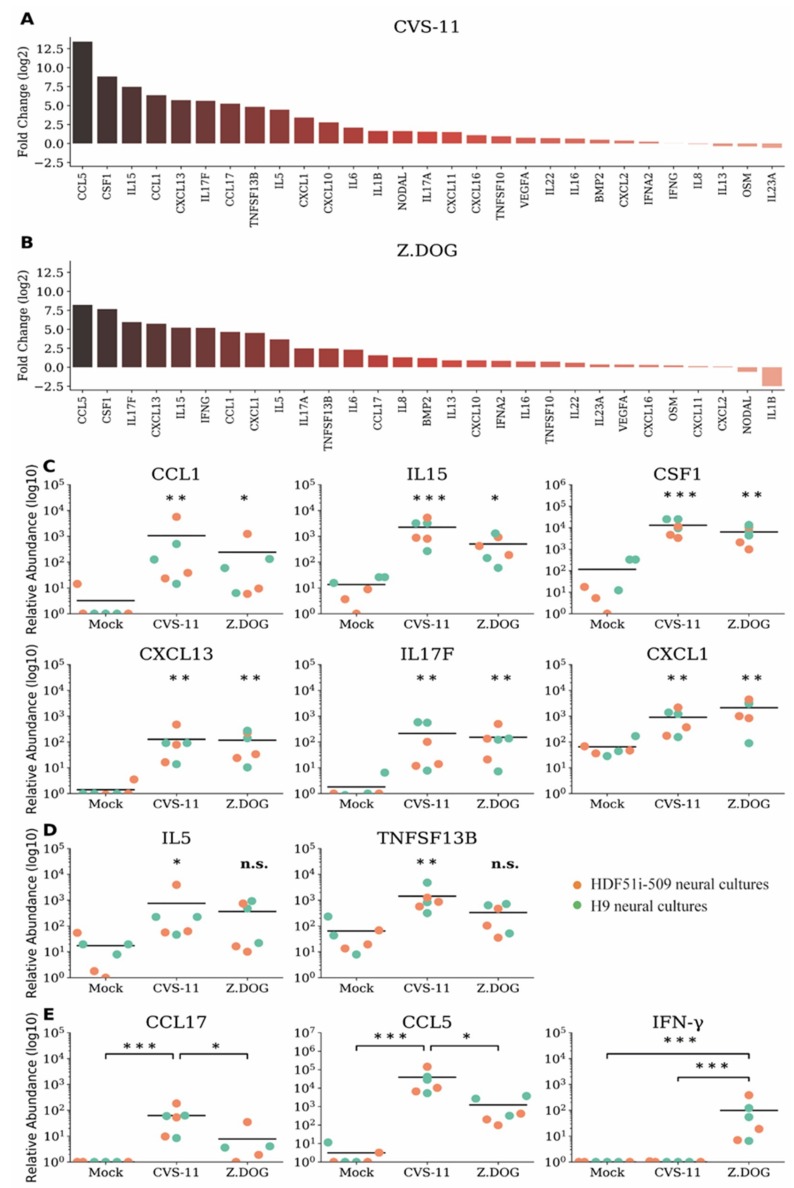
Gene expression profiles of chemokines and cytokines in H9- and HDF51i-509-derived human neural cultures infected with CVS-11 and Z.Dog rabies virus. H9-NPCs and HDF51i-509-NPCs were differentiated for 27 days and 28 days, respectively, before infection with rabies virus. qPCR analysis of chemokine and cytokine genes (29 genes selected from initial screen of 91 genes, Supplementary Materials Table S1) in H9-NPC and HDF51i-509-NPC-derived neural cultures infected with CVS-11 and Z.Dog rabies virus for 72 h at virus:cell ratio of 1. (**a**,**b**) Graphs show fold change (log2) of all 29 genes screened relative to mock infection in both H9-NPC-derived and HDF51i-509-NPC-derived neural cultures. (**a**) Infection with CVS-11 and (**b**) infection with Z.Dog strain. (**c**) Graphs show group of cytokines found to be significantly upregulated (*p* < 0.05) in both CVS-11 and Z.Dog infection, when compared with mock infection. (**d**) Graphs show IL5 and TNFSF13B/BAFF gene expression levels which were found to significantly regulated in CVS-11 infection but not with Z.Dog. (**e**) Graphs show group of cytokines including IFN-γ found to differently upregulated in Z.Dog infection in comparison to CVS-11 infection. * *p* < 0.05, ** *p* < 0.01, *** *p* < 0.001, n.s. = not significant. *n* = 3. Mean values are indicated by horizontal lines in the graph.

We next examined whether rabies infection of human stem cell-derived neural cultures with rabies virus could trigger an immune response despite the lack of neuronal apoptosis. In this study, we first analyzed the gene expression profiles of a comprehensive list of chemokines and cytokines (Supplementary Table S1) in H9-NPC-derived neural cultures infected with CVS-11 and Z.Dog strains of rabies virus. In this initial analysis, we shortlisted 29 differentially expressed cytokine and chemokine genes (Supplementary Table S1), for which a custom array of primers was designed for further qPCR analysis. These gene expression profiles were then screened in neural cultures derived from both H9-NPCs and HDF51i-509-NPCs, infected with CVS-11 (Figure 4a) and Z.Dog (Figure 4b) rabies virus. Gene expression profiles of chemokines and cytokines were quantified by the relative fold change compared to mock-infected neural cultures treated with PBS (Figure 4a,b) and their relative transcript abundance (Figure 4c–e). In this analysis, the highest upregulation was observed for CCL5/RANTES and CSF1 in both CVS-11 (Figure 4a) and Z.Dog rabies infection (Figure 4b), compared with mock infection. Other proinflammatory genes which were significantly upregulated in both the rabies virus strains include IL15, IL17F, CCL1, CXCL13, and CXCL1 (Figure 4c). In addition, IL5 and TNFSF13B/BAFF were found to be significantly upregulated in CVS-11 infection but not with the Z.Dog strain (Figure 4d). CCL17 and CCL5 mRNA were both upregulated at significantly higher level in CVS-11 infection compared to Z.Dog infection (Figure 4e). Interestingly, while we did not observe a significant upregulation of type I interferon gene (IFNA2) in neither CVS-11 nor Z.Dog infection, we observed specific upregulation of type II interferon gene (IFN-γ) in Z.Dog infection (Figure 4e). The expression of this IFN-γ gene was undetectable in the mock infected or CVS-11 infected neural cultures (Figure 4e). These results show a robust but strain specific immune response in both H9-NPC- and HDF51i-509-NPC-derived neural cultures in response to rabies infection.

### 3.5. Stem Cell-Derived Ex-Vivo Models of Human Neuronal Network Reveals Differential Dynamics in the Axonal Transmission of Rabies Virus Strains

**Figure 5:**
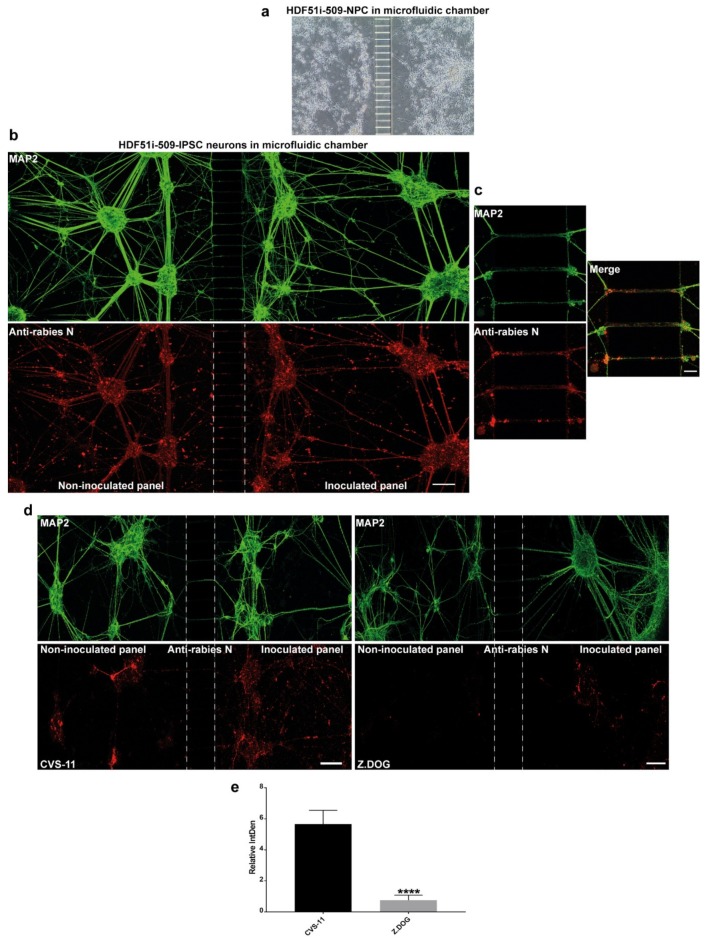
Stem cell-derived ex-vivo model of human neuronal network. (**a**) Representative DIC image taken using 10× objective of Xona microfluidic device seeded with HDF51i-509-NPCs at 5 days post-differentiation. (**b**) HDF51i-509-NPCs after 26 days of differentiation in microfluidic device were infected with CVS-11 rabies virus in the inoculated panel for 72 h. A unidirectional flow of media from the non-inoculated panel to the inoculated panel is maintained to prevent random diffusion of viral particles. Immunostaining with MAP2 (green) reveals extensive neuronal network between the panels after 26 days of differentiation. Immunostaining with rabies nucleoprotein antibody (red) shows equivalent viral antigen between both panels, indicating efficient spread of CVS-11 rabies infection within and between the neuronal networks of both panels. Confocal images were taken using 20× objective with tile function and stitched together. Scale bar 100 µm (images are maximum intensity projection of Z-stacks) (**c**) Representative high magnification (40×) confocal images shown in (**b**). Scale bar 10 µm. Images show spread of rabies antigen (red) in MAP2-neurites (green) within microchannels. (**d**) HDF51i-509-NPC-derived neural cultures in microfluidic device after 26 days of differentiation were infected with CVS-11 or Z.Dog rabies virus for 24 h. Confocal images show increased presence of rabies antigens (red) in the non-inoculated panel of CVS-11 infection when compared with Z.Dog infection. (**e**) Quantification of relative intensity of rabies nucleoprotein staining in the inoculated panel versus non-inoculated panel in CVS-11 and Z.Dog infection. Data show significantly reduced viral antigen staining in the non-inoculated panel of Z.Dog infection compared to CVS-11 infection. **** *p* < 0.0001 Z.Dog versus CVS-11 infection, *n* = 3.

Axonal transmission of rabies virus between trans-synaptically connected neurons in the host nervous system is an important feature of rabies pathogenesis. However, this mechanism has not been studied in human neurons. In this study, we used a microfluidic chamber to generate a human ex-vivo neuronal model system for studying axonal transmission of rabies virus between interconnected neurons. Here, we seeded hNPCs derived from HDF51i-509 in the adjoining panels of a microfluidic device separated by microchannels which prevents the passage of cell bodies (Figure 5a). When these hNPCs were differentiated as previously described, the developing axonal structures passed through the microchannels, connecting the neuronal cultures on adjacent panels. After 26 days of hNPC differentiation, the neural cultures were infected with CVS-11 rabies virus on the inoculated panel and fixed after 72 h of infection in the microfluidic device. In such infections, a higher volume of media is maintained in the non-inoculated panel to maintain unidirectional flow of culture media and avoid random diffusion of viral inoculum from the inoculated panel, except via axonal transmission. Immunostaining and confocal microscopy analysis of these neural cultures displayed extensive detection of MAP2-postive neuronal networks between the panels (Figure 5b). Immunostaining with rabies nucleoprotein antibody also revealed efficient axonal transmission of rabies virus from the infected neurons in one panel to the uninfected neurons in the other panel after 72 h of infection (Figure 5b,c). We further investigated any difference in axonal transmission between the CVS-11 and Z.Dog strains in this model system. HDF51i-509-NPCs were similarly differentiated in microfluidic chambers and inoculated with either CVS-11 or Z.Dog strains. Following 24 h inoculation, the cultures were fixed and analyzed by immunostaining. This analysis identified extensive infection with CVS-11 rabies virus strain in the inoculated panel as well as in the non-inoculated panel, suggesting axonal transmission of the virus extensively within the neuronal network within 24 h of infection (Figure 5d). Whilst the Z.Dog strain was able to infect the MAP2-positive neurons in the inoculated panel, the spread of infection to neurons in the non-inoculated panel was significantly reduced compared to CVS-11 (Figure 5d,e). These results suggest that there may be strain specific differences in the dynamics of viral replication and axonal transmission of rabies virus in human neuronal network.

## 4. Discussion

In this study, we demonstrate that human ESC- and iPSC-derived neural cultures could be used as effective ex-vivo models to study several pathogenic mechanisms associated with human rabies. We generated ESC- and iPSC-derived human neural cultures consisting of mature neurons identified by positive immunostaining for mature neuronal markers and development of calcium signaling mechanisms. We show that these ESC- and iPSC-derived neural cultures are highly susceptible to infection with different strains of lyssavirus. We then sought to perform a basic validation of whether this system could reproduce key features of rabies pathogenesis, which have been previously identified in classical in-vitro and in-vivo mouse model systems. Lyssavirus infection in human neural cultures did not led to the induction of apoptosis but resulted in a pro-inflammatory state as evidenced by upregulation of specific subsets of cytokine genes, consistent with the findings in mouse models [23,25,26,30,81,82,83]. In addition, we demonstrate the need to consider differences in the characteristics of viral strains when selecting ex-vivo models for human rabies. We show strain-specific difference in the cytokine upregulation and axonal transmission of rabies virus between interconnected axons in our novel system of synaptically connected human neurons. In summary, these results show that human stem cell-derived neural cultures could be used as effective models to study the cellular and molecular pathogenic mechanisms of human rabies, alongside classical animal models.

The evasion of host immune response is a key feature of rabies pathogenesis. The viral mechanisms behind immune evasion are yet to be investigated at a cellular level in human neural culture systems. Therefore, to validate whether our model could be suitable for this objective, we examined cytokines and chemokine response in stem cell-derived human neural cultures infected with rabies virus. Most of the cytokines identified to be upregulated in this study have demonstrated roles in mediating neuroinflammation by promoting infiltration and activation of immune cells into the CNS, activation of CNS resident inflammatory glial cells, and increased permeability of BBB. CCL5 was found to be the highest upregulated chemokine gene in human neural cultures in response to infection with both CVS-11 and Z.Dog rabies infection compared to uninfected cultures. CCL5 plays a key role in the disruption of BBB leading to the infiltration of inflammatory cells in the CNS and neuroinflammation in various pathological conditions [84,85,86,87,88]. Previous studies in mice have also identified CCL5 to be the highest upregulated chemokine in response to both attenuated and wildtype rabies virus infection in-vivo [25]. Additionally, inhibiting CCL5 signaling prolonged survival time of mice infected with rabies virus, by reducing the upregulation of proinflammatory cytokines in the CNS [25]. These findings, in addition to our data, suggest that CCL5 may be a target for therapeutic intervention in human rabies. However, CCL5 plays a role in controlling neurotransmission by modulating glutamate release at the synapses [89,90] and dysregulation of CCL5 mediated glutamatergic transmission is linked with psychiatric disorders [89,90,91,92]. In addition, CCL5 also promotes neuronal survival during injury involving toxic insults [93,94]. Hence, a broader investigation of CCL5 in rabies pathogenesis is necessary to fully understand its role beyond classical proinflammatory signaling in the CNS and scrutiny needs to be exercised when considering a therapeutic option that may aggressively target these pathways. The implications of comparatively reduced upregulation of CCL5 in non-adapted Z.Dog rabies infection also needs to be studied in this model system.

CSF1 was found to be the second most upregulated chemokine in response to both CVS-11 and Z.Dog strain rabies infection in human neural cultures. CSF1 regulates microglial activation and proliferation [95] and also plays a role in local activation of infiltrating T cells aiding viral clearance from the brain [96]. In concert with CSF1 upregulation, other cytokines involved in microglial activation and proliferation, such as CCL1 [97] and IL15 [98], were found to be upregulated in both CVS-11 and Z.Dog strain infections. In addition, other microglia activating cytokines, such as IL5 [99] and CCL17 [100], were found to be significantly upregulated in CVS-11 infection. Cytokines relating to the activation of non-CNS resident immune cells were also found to be upregulated in this study, including CXCL13 (B cells) [101] and CXCL1 (neutrophils) [102]. B-cell activating factor (BAFF) encoded by the TNFSF13B gene, which mediates the proliferation of B cells in the CNS [103], was found to be significantly upregulated in CVS-11 infection. In contrast, we identified specific upregulation of type II interferon IFN-γ in Z.Dog rabies virus infection, which was not detected in uninfected and CVS-11 rabies virus infected neural cultures. IFN-γ activates a potent anti-viral response through STAT1 signalling [104,105] and also functions in altering tight-junction proteins, leading to increased BBB permeability [27,106]. In summary, rabies infection in human neuronal cultures results in the upregulation of specific cytokines involved in neuroinflammation by activation of microglia and other infiltrating immune cells in the CNS. The mechanisms behind upregulation of these cytokines, downstream cascades, or potential viral inhibition of cytokine-induced responses were not examined in this study. None the less, the observation of specific cytokine response in this novel human stem cell-derived neuronal model systems following rabies infection, demonstrates that this culture system could serve as an effective tool for future investigation of host viral interactions.

In addition to specific cytokine response, the human stem cell-derived neural cultures developed in this study also demonstrated transsynaptic transmission of rabies virus, a key mechanism responsible for its spread throughout the nervous system [5]. Rabies virus interacts with neuronal membrane receptors and hijacks endosomal trafficking pathways in the axons to mediate anterograde transport in the nervous system [10,35,107]. The reverse genetics approach has enabled the identification of several viral elements responsible for neuroinvasion and axonal trafficking [10,35,108]. In this study, we have developed a stem cell-derived ex-vivo model of human neuronal network which enables the study of viral replication and axonal trafficking between human neuronal populations. We further demonstrate more efficient virus replication and axonal transmission in a laboratory-adapted CVS-11 strain compared to a non-adapted Z.Dog strain in this model, consistent with existing knowledge [108,109]. Therefore, this newly described infection model provides an enhanced capability to investigate molecular mechanisms of rabies axonal trafficking in human neurons, using techniques such as reverse genetics.

In summary, this study introduces a new ex-vivo model system for studying the key pathogenic features of rabies in human neurons. This ex-vivo model system provides exciting new opportunities in identifying novel cellular mechanisms associated with the pathogenesis of rabies and will compliment current in-vitro and in-vivo models for studying this devastating disease.

## Supplementary Materials

The following are available online at https://www.mdpi.com/1999-4915/12/4/359/s1, Table S1: List of 91 genes initially screened by qPCR in total RNA extracted from H9-NPC-derived neural cultures infected with CVS-11 and Z.Dog strains. Table S1 highlights the genes selected for custom array qPCR assay with corresponding CT values. Video S1: Representative video from calcium imaging experiments. H9-NPCs were differentiated for 21 days and imaged with calcium-binding Fura-2 AM dye. Video S1 shows response of neural cultures upon stimulation with 100 µM ATP.
