# Supplementary material for: Modelling Lyssavirus Infections in Human Stem Cell-Derived Neural Cultures
Source: Viruses. 2020 Mar 25;12(4):359. doi: 10.3390/v12040359 (PMC7232326; doi:10.3390/v12040359)
Supplement: Supplementary file 1 [file viruses-12-00359-s001.zip › Suppl. Table 1.pdf]

	CT values		
Targets	Uninfected	CVS-11	Z.DOG
ACTB	19.10	19.41	18.74
CCL1	0.00	34.45	34.97
CCL21	27.18	27.32	27.25
CNTF	26.33	26.36	26.27
CXCL12	27.62	27.97	27.46
GUSB	26.68	27.01	26.47
IL15	34.03	30.23	31.72
IL2	34.71	35.59	34.72
IL5	35.00	32.77	33.17
MIF	21.05	20.95	20.59
THPO	27.84	27.31	27.31
TBP	26.28	25.96	26.06
ADIPOQ	27.32	26.96	26.92
CCL11	27.09	26.98	26.96
CCL22	33.79	35.43	38.83
CSF1	26.37	21.09	21.54
CXCL13	0.00	34.52	34.26
IFNA2	27.33	26.97	26.97
IL16	31.97	30.83	30.46
IL21	0.00	0.00	34.87
IL6	29.20	28.05	27.81
MSTN	29.28	30.61	30.01
TNF	27.69	27.27	27.18
GADPH	19.50	19.67	19.19
B2M	21.43	19.28	20.25
CCL13	0.00	0.00	0.00
CCL24	26.78	26.77	26.61
CSF2	27.07	26.98	27.08
CXCL16	25.37	23.33	24.61
IFNG	0.00	0.00	34.93
IL17A	37.89	35.00	35.58
IL22	34.78	34.37	33.14
IL7	26.94	26.30	26.62
NODAL	36.59	34.42	35.32
TNFRSF11B	25.75	26.09	26.18
HPRT1	28.24	28.52	28.20
BMP2	33.72	32.99	32.85
CCL17	0.00	35.23	35.13
CCL3	25.84	25.58	25.58
CSF3	27.82	27.57	27.61
CXCL2	28.84	28.21	28.18
IL10	26.91	26.69	26.81
IL17F	38.17	35.35	34.77
IL23A	34.23	33.00	33.33

IL8	26.98	26.84	26.74
OSM	27.35	26.95	26.81
TNFSF10	27.04	24.78	26.33
gDNA	27.09	26.97	27.11
BMP4	26.31	25.73	25.94
CCL18	26.77	26.83	26.69
CCL5	34.48	25.92	29.32
CX3CL1	25.94	25.76	25.98
CXCL5	26.79	26.90	26.82
IL11	27.93	27.32	27.30
IL18	34.01	33.52	33.31
IL24	35.26	35.55	34.63
IL9	0.00	0.00	0.00
PPBP	27.14	27.15	27.12
TNFSF11	35.50	34.52	34.52
PCR	35.52	34.56	35.30
BMP6	32.36	34.15	31.92
CCL19	27.35	27.39	27.30
CCL7	34.64	34.33	35.52
CXCL1	32.52	31.03	31.14
CXCL9	0.00	30.83	32.99
IL12A	31.20	30.70	30.95
IL1A	32.10	31.40	31.27
IL27	0.00	37.69	35.48
LIF	27.35	27.16	26.81
RPLPO	22.19	23.34	22.54
TNFSF13B	35.01	30.00	31.94
RQ1	22.11	20.18	21.28
BMP7	24.81	26.34	25.17
CCL2	23.67	25.38	24.19
CCL8	26.95	26.97	26.94
CXCL10	26.39	23.17	25.34
FASLG	26.39	26.69	26.27
IL12B	34.52	34.79	0.00
IL1B	35.05	34.53	33.35
IL3	0.00	0.00	35.37
LTA	27.03	26.98	26.99
SPP1	27.19	26.86	26.51
VEGFA	23.86	22.85	22.36
RQ2	22.97	20.79	21.95
C5	28.23	28.34	28.21
CCL20	0.00	32.13	32.22
CD40LG	35.58	38.13	37.11
CXCL11	26.80	24.46	25.95
GPI	23.28	23.53	22.90
IL13	29.34	28.56	28.91

IL1RN	35.54	35.24	32.91
IL4	38.06	39.68	0.00
LTB	29.98	30.19	30.33
TGFB2	22.46	0.00	23.42
XCL1	25.33	0.00	0.00
